# Diversifying crop rotations with pulses enhances system productivity

**DOI:** 10.1038/srep14625

**Published:** 2015-10-01

**Authors:** Yantai Gan, Chantal Hamel, John T. O’Donovan, Herb Cutforth, Robert P. Zentner, Con A. Campbell, Yining Niu, Lee Poppy

**Affiliations:** 1 Semiarid Prairie Agricultural Research Centre, Agricultural & Agri-Food Canada, Gate#3, Airport Road East, Swift Current, Saskatchewan, S9H 3X2; 2 Lacombe Research Centre, Agriculture & Agri-Food Canada, 6000 C&E Trail, Lacombe, Alberta, T4L 1W1; 3 Eastern Cereal and Oilseed Research Centre, Agriculture and Agri-Food Canada, 960 Carling Ave., Ottawa, Ontario, K1A 0C6.; 4 Gansu Provincial Key Laboratory for Aridland Crop Science, Gansu Agricultural University, Lanzhou, 730070, China

## Abstract

Agriculture in rainfed dry areas is often challenged by inadequate water and nutrient supplies. Summerfallowing has been used to conserve rainwater and promote the release of nitrogen via the N mineralization of soil organic matter. However, summerfallowing leaves land without any crops planted for one entire growing season, creating lost production opportunity. Additionally, summerfallowing has serious environmental consequences. It is unknown whether alternative systems can be developed to retain the beneficial features of summerfallowing with little or no environmental impact. Here, we show that diversifying cropping systems with pulse crops can enhance soil water conservation, improve soil N availability, and increase system productivity. A 3-yr cropping sequence study, repeated for five cycles in Saskatchewan from 2005 to 2011, shows that both pulse- and summerfallow-based systems enhances soil N availability, but the pulse system employs biological fixation of atmospheric N_2_, whereas the summerfallow-system relies on ‘mining’ soil N with depleting soil organic matter. In a 3-yr cropping cycle, the pulse system increased total grain production by 35.5%, improved protein yield by 50.9%, and enhanced fertilizer-N use efficiency by 33.0% over the summerfallow system. Diversifying cropping systems with pulses can serve as an effective alternative to summerfallowing in rainfed dry areas.

Agroecosystem productivity is often constrained by a low availability of water and nutrients^1^, and the challenge is serious in many arid and semiarid regions of the world, such as Southwest Australia^2^, Northwest China^3^, northern Eurasia^4^, central Africa^5^, and the northern Great Plains of North America^6^. To tackle these challenges, many approaches have been employed, but summerfallow has been historically used as one of the mainstream farming practices in these dry areas. For example, in the mid-1970 s, approximately 11 million hectares of farmland were in summerfallow on the Canadian prairies alone, accounting for approximately 40% of the total annual crop land of the region; the area of summerfallow has declined substantially in recent years, but approximately 3.5 million hectares of land remained in summerfallow by 2013^7^. Summerfallowing leaves land unplanted for one entire growing season, during which a proportion of the rainfall can be conserved in the soil profile^8^,^9^, which is then available for crops grown the following year^10^. Additionally, summerfallowing encourages the release of nitrogen (N) via the N mineralization of soil organic matter^11^, thus increasing soil N availability and helping to reduce the amount of inorganic N-fertilizer in farming systems^12^.

However, a growing body of evidence has shown that summerfallowing has serious environmental consequences^13^. Tillage during the summerfallow period disturbs the soil, encourages soil erosion^14^, and generates dust^13^ that affects soil, air and water quality^15^. Tillage and herbicides for weed control during summerfallow use fossil fuels^16^ that emit greenhouse gases, thereby contributing to climate change^17^. Frequent summerfallowing causes serious soil degradation over the years^18^, as tillage facilitates crop residue decomposition and accelerates the loss of soil organic matter^19^. Furthermore, frequent summerfallowing in crop rotation systems increases the carbon footprint of agriculture^20^. A question that is frequently asked is whether the two main attractive features of summerfallow (i.e., conserving rainwater and providing soil N benefits) can be retained using alternative strategies without or with minimal environmental impact.

In searching for alternative, non-summerfallowing farming strategies, we determined that diversifying cropping systems with annual pulse crops, such as dry pea (*Pisum sativum* L.), lentil (*Lens culinaris* Medikus), and chickpea (*Cicer arietinum*) could increase the systems’ productivity while decreasing the environmental impact. The inclusion of pulse crops in farming systems can enhance soil available N^21^ due to the ability of pulse plants to fix atmospheric N_2_ through symbiosis with *Rhizobium*^22^. In many areas of Mediterranean countries, the use of pulses to enhance soil N has been practiced for decades, and the advantages have been widely demonstrated^23^,^24^. However, it is not known whether diversifying summerfallow systems with pulses is effective and productive in the northern latitude areas where water is scarce and the growing season is short (95 to 125 days)^25^. Our proposal for diversifying summerfallow systems with short-season pulses is largely based on the latest research on pulses: (**a)** pulse plants in the northern latitudes have a shallow rooting depth^26^ with approximately 77–85% of the roots being located in the 0–0.4 m soil depth^27^, which allows pulse crops to use water mainly from the top 0.6 m soil layer, leaving water in the deeper soil layers (below 0.6 cm)^28^ for use by deeper-rooted crops that are grown the following year^29^; (**b**) dry pea and lentil, the two main annual pulses grown in the semiarid northern Great Plains, use 15–35% less water than cereal or oilseed crops, thereby enhancing water use efficiency^30^; (**c**) pulses are typically harvested several weeks earlier than cereal or oilseed crops, leaving a longer postharvest period during which soil water can accumulate prior to planting crops the following spring^31^; (**d**) the inclusion of pulse crops in the rotation can increase crop yields, decrease inputs of inorganic N fertilizer^32^, and enhance N use efficiency^33^; and finally (**e**) long-term studies have shown that crop diversification with pulses and oilseed can improve overall farming sustainability^6^.

The central hypothesis of the present study is that diversifying cropping systems with pulse crops can improve the attributes of soil water conservation and soil N benefits and increase total grain production. To test the hypothesis, we conducted a 3-yr crop sequence study that was repeated five times (i.e., five cycles) from 2005 to 2011 (Table 1). In the 3-yr cropping sequences, spring wheat (*Triticum aestivum* L.) was grown in Year-1, followed by crops of various pulses in Year-2, a cereal [spring wheat or barley (*Hordeum vulgare)*], and a summerfallow control. In Year-3, durum wheat (*Triticum turgidum* subsp. *durum*) was planted on the different stubble generated from the Year-2 crops and on the summerfallow control. In each of the five cycles, we measured soil water content and soil N status at three key stages each year: (i) water and N remaining in the different soil layers at the harvest of Year-2 crops, (ii) the additional soil water and N gained (or otherwise lost) over the postharvest (fall and winter) periods, and (iii) the total soil water and soil N available at the planting of the Year-3 crop (durum wheat). These detailed measurements provide strong confidence in our ability to assess the effectiveness of diversifying cropping systems with pulses in terms of their effects on soil water, soil N status, and total grain production over an entire cropping cycle.

## Results

### Diversifying cropping systems with pulses improves soil water use

#### First—Water remaining in the soil profile at harvesting the Year-2 crops

**Figure 1:**
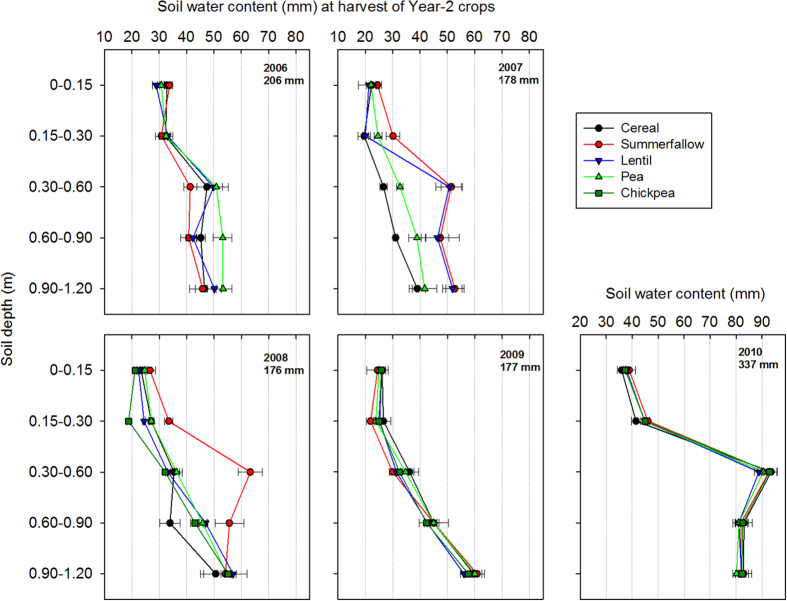
Soil water remaining at the various depths of the 0–1.2 m soil profile at the harvest of the Year-2 crops. The Year-2 crops were dry pea, lentil, chickpea, and a cereal (spring wheat or barley) that were no-till planted in the field of Year-1 wheat stubble in each of the five cycles (summerfallow was the control). The lines at each point are the standard errors of the means (n = 4).

We found substantial variations in the quantity of water remaining in the soil profile during the test years (Fig. 1). An overall average of approximately 180 mm of water remained in the soil profile to a depth of 1.2 m for cycles-2 (2007), -3 (2008), and -4 (2009), and approximately 206 mm remained in cycle-1 (2006), and 337 mm in cycle-5 (2010). A portion of the remaining soil water was above the ‘permanent wilting point’ which is an indicator of the minimal soil moisture the plant requires not to wilt. At the experimental site, the permanent wilting point of the water content was 134 mm^34^, and water content greater than 134 mm should be available to the Year-3 durum wheat. Generally, in cycles-1 (2006), -4 (2009) and -5 (2010), the soil under summerfallow contained similar amounts of water at the harvest of the Year-2 crops as the cropped fields. However, in cycles-2 (2007) and -3 (2008), the soil water content in the 0–1.2 m depth was approximately 53 mm more in summerfallow compared to cropped fields, which corresponded to an increase of 32%.

The water distribution profile across the 1.2 m rooting zone showed that the amount of water remaining in the various soil layers generally increased with soil depth, with the absolute values varying each year (Fig. 1). For example, in 2006 the top 0–0.15 and 0.15–0.30 m soil depths each had less than 32 mm of water remaining at the harvest of the Year-2 crops, whereas in the 0.60–0.90 and 0.90–1.2 m depths, 47 mm water remained. In 2009, the water remaining in the 0.30–0.60 m depth (91 mm) was nearly triple the amount of water remaining in the top 0.15 m soil layer. Summerfallow and the cropped lands had similar soil water distribution patterns across the rooting zone in 2006, 2009, and 2010 (Fig. 1). However, in 2007, the summerfallow fields had 12% more water remaining in the top 0–0.15 and 41% more remaining in the 0.15–0.30 m soil layers than the cropped plots; in 2008, summerfallow had 17%, 39%, 87%, and 25% more water remaining in the four soil depths, respectively, than the cropped lands. Notably, the lentil fields had similar soil water content as summerfallow in three lower depths, and they were 57%, 22% and 26% greater, respectively, than the soil water content that was found in the corresponding depths of the cereal crop.

#### Second—Water recharged to the soil profile post harvesting the Year-2 crops

The duration from the harvest of the Year-2 crops to the planting of the Year-3 durum wheat the following spring was approximately 7–9 months, allowing time for precipitation to infiltrate, redistribute and recharge the rooting zone. The amount of water gained through the recharge process varied between soil depths and varied each year (Table 2). On average, the water gained in this process was 18%, 46%, 23%, and 27% of the soil water present when planting the Year-3 durum wheat in 2006–07, 2007–08, 2008–09, and 2009–10, respectively. In spring 2010, however, approximately 34% of the water remaining in the previous fall was lost (most likely through evaporation or draining to depths lower than 1.2 m). The differences between the summerfallow and cropped fields in recharging the soil profile varied each year. From fall 2007 to spring 2008, the cropped fields were recharged with 57% more water than the summerfallow, whereas in fall 2008 to spring 2009, there was no difference in the recharge amount between the summerfallow and the cropped fields. However, the opposite response occurred in fall 2006 to spring 2007 and in fall 2009 to spring 2010, where the summerfallow fields were recharged with 66 mm and 58 mm of water, respectively, which was greater than the amount of recharge for the cropped fields which was 16 mm and 30 mm, respectively. In contrast to the other years, in fall 2010 to spring 2011, all fields lost a significant amount of water, ranging from a loss of 50 mm from the barley fields, to 67 mm from the summerfallow control, and 80 mm from the chickpea fields.

#### Third—Total amount of water in the soil profile at the planting of the Year-3 durum wheat

**Figure 2:**
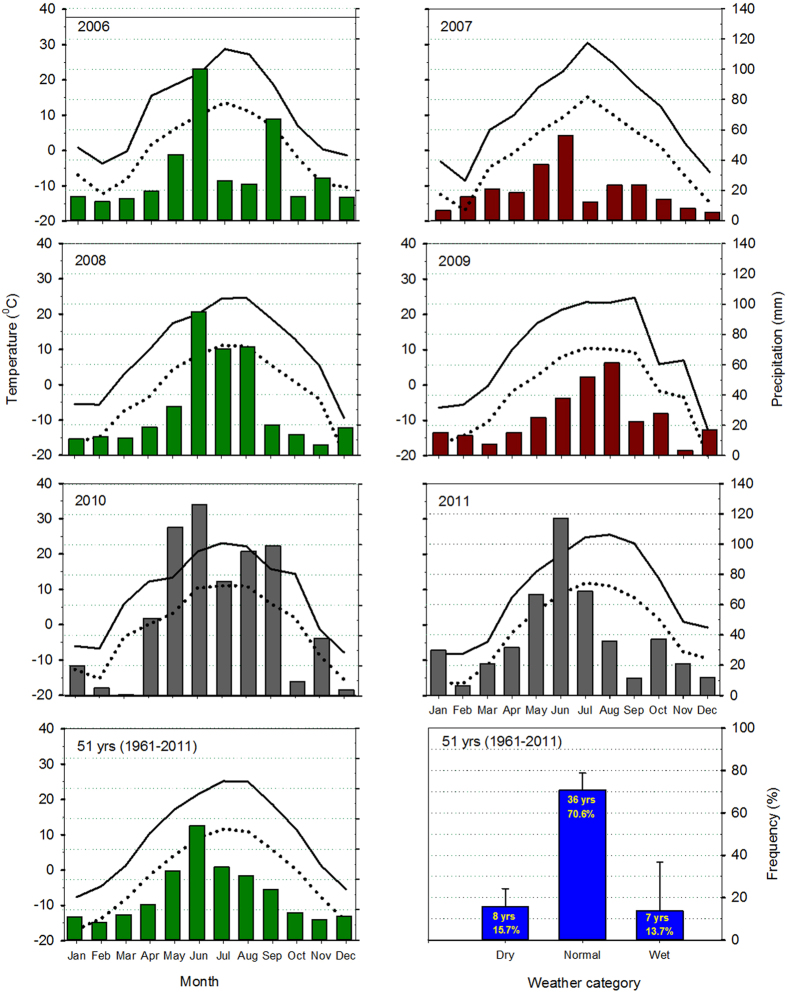
Weather conditions during the course of the field experiment. The monthly maximum and minimum air temperatures and precipitation in each year are compared with the long-term (1961–2011) averages. The frequency of the dry, normal, and wet years is based on the recent 51 years of records in Swift Current, Saskatchewan, Canada.

On average, the total amount of water to a depth of 1.2 m at planting the Year-3 durum wheat varied from 215 mm in 2008 to 270 mm in 2011 (Table 2). There were differences in the total amount of water between treatments, but the ranking of the differences varied largely between test years. The large variation between years was a reflection of the quantity of water remaining at the harvest of the previous crops and the postharvest water recharge activity. For example, the low amount of water at planting in 2009 was largely due to the limited precipitation between fall 2008 to spring 2009 (Fig. 2), whereas the high soil water content at planting in 2011 was due to very high water content in the deeper soil layers that remained at the harvest of the 2010 crops (Fig. 1).

### Diversifying cropping systems with pulses improves soil N availability

**Figure 3:**
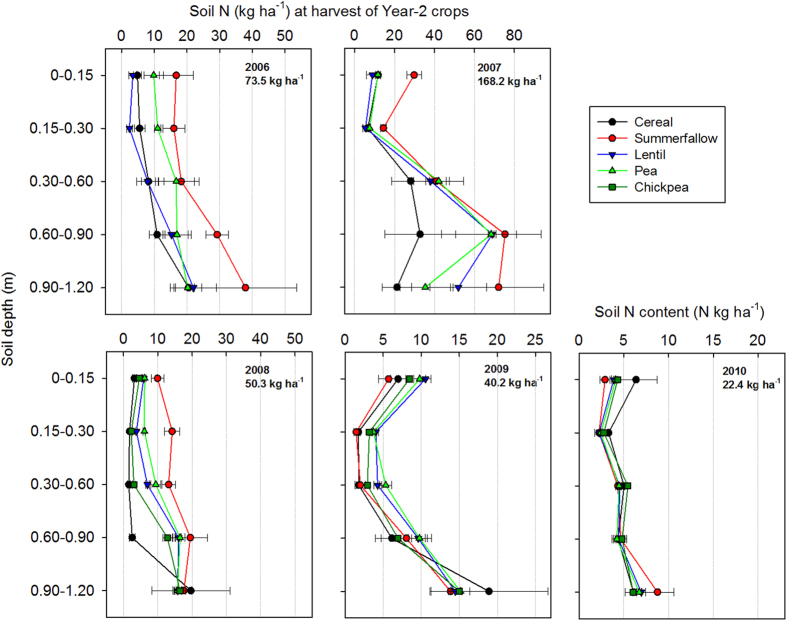
Residual soil N at the various depths of the 0–1.2 m soil profile measured at the harvest of Year-2 crops. The Year-2 crops were dry pea, lentil, chickpea, and a cereal (spring wheat or barley) that were no-till planted in the field of Year-1 wheat stubble in each of the five cycles (summerfallow was the control). The lines at each point are the standard errors of the means (n = 4).

The quantity of mineral soil N (NO_3_^−^ plus exchangeable NH_4_^+^) remaining in the 0–1.2 m soil depth at the harvest of the Year-2 crops varied largely among the crops and test years (Fig. 3). In 2006, 2007, and 2008, the fields with pulses had 63.2, 169.1, and 47.4 kg ha^−1^ of soil N remaining at harvest, respectively, which were 26, 68, and 65% greater than the fields with the cereal; similarly, the summerfallow control had 137, 131, and 160% more soil-N remaining compared to the fields with the cereal. The differences between treatments were observable across the 0–1.2 m rooting zone, with the largest differences occurring in the topsoil layers. In 2006, 2007 and 2008, soil N was higher for the summerfallow fields compared to the cropped fields, whereas the opposite occurred in 2009 when the fields with pulses had 33% more N remaining at harvest than the summerfallow control and 16% more N than the field after the cereal. In 2010, there was no significant difference between treatments, with an average of soil N of 22.4 kg ha^−1^ across the 0–1.2 m rooting zone (Fig. 3).

The soil N status changed from the harvest of the Year-2 crops to the planting of the Year-3 durum wheat the following spring, and the magnitude of the changes varied among the soil depths and test years (Table 3). From fall 2006 to spring 2007, all fields lost an average of 30.8 kg ha^−1^ of N with the loss occurring at all soil depths. From fall 2007 to spring 2008, the summerfallow field lost 63.9 kg ha^−1^ of N, whereas the fields after pulse crops gained 25.3 kg ha^−1^ of N, and the field after cereal gained 18.5 kg ha^−1^. In the last three periods (2008–09, 2009–10, and 2010–11), all the fields had an increased amount of N. Across the three latter years (2008–09, 2009–10, 2010–11), the average gain of soil N from the previous harvest to the following spring was 13.6 kg ha^−1^, and the differences between treatments were inconsistent from year to year.

There was a similar trend for the treatment effects on residual soil N at the planting time of the Year-3 durum wheat during the five study cycles (Table 3). Each cycles, the pulse system and the summerfallow control had significantly higher soil N at the planting of the durum wheat than the cereal-monoculture system (*P* < 0.05; n = 5 cycles × 4 replicates). When averaged over the five cycles, the available soil N in the 0–1.2 m soil profile at the planting of the durum wheat was 76.3 kg ha^−1^ in the fields following pulses, which was 57.5% greater compared to the field following the cereal. However, in four of the five cycles, spring soil N in the 0–1.2 m depth was 15.1% lower in the fields following pulses compared to the summerfallow fields.

### Diversifying cropping systems with pulses increases crop production

The preceding crop or summerfallow had significant effects on the grain yield, protein yield (a product of grain dry weight by protein concentration), and the N dynamics of durum wheat grown in the Year 3 of the cropping sequence, and the magnitude of the effect varied each year (Table 4). In 2008, 2010, and 2011 with normal to above-normal precipitation, the durum wheat that was grown on pulse stubble produced a similar grain yield as the durum wheat that was grown on the summerfallow. In 2011, the durum wheat that was grown on chickpea stubble produced a significantly greater (19%) yield than the durum wheat that was grown on the summerfallow, and nearly double the grain yield of the durum wheat that was grown on barley stubble. However, in the drier years of 2007 and 2009, the durum wheat grown on the summerfallow produced 36.5% more grain than the crop that was grown on pulse stubble. The durum wheat that was grown on pulse stubble produced 27.8% more grain than the crop that was grown on barley stubble.

The grain crude protein concentration of durum wheat varied significantly among the test years, averaging 153.1, 128.4, 142.2, 117.6 and 99.5 g kg^−1^, in 2007, 2008, 2009, 2010 and 2011, respectively. These values changed minimally with treatments. As a result, the effect of the preceding crops (summerfallow) on protein yield followed a similar pattern as the effect on grain yield (Table 4). Averaged across the five cycles, the durum wheat preceded by various pulse stubbles had an average protein yield that was 72.8% greater compared to the durum wheat preceded by the cereal, but it was 13.1% lower compared to the durum wheat preceded by the summerfallow.

The total N uptake by durum wheat varied from a minimum of 45.8 N kg ha^−1^ in 2010 to a maximum of 72.7 N kg ha^−1^ in 2008 (Table 4), with N uptake in the seed accounting for 85.1% of the total N uptake. On the basis of plant N uptake, soil N available at pre-planting, soil N remaining at crop harvest, and N applied to the crop through fertilizers, we estimated the potential mineralized-N during the durum wheat growth period. Across an average of the five cycles, the values of potential mineralized-N varied from −3.9 to 84.9 N kg ha^−1^ (Table 4). The fields following the cereal and pulses had an equivalent value of N mineralization during the durum wheat growth period: both were 51.8% greater compared to the durum wheat growing fields following the summerfallow control.

The choice of cropping systems had a significant impact on total grain production over a 3-yr cropping cycle (i.e., grains produced by Year-1 plus Year-2 plus Year-3 crops). Under dry conditions, the wheat-pulse-durum system produced 36.7% more grain yield and 61% more protein yield per 3-yr cycle than the wheat-summerfallow-durum system (Table 5). With the total amount of fertilizer applied in the two systems being similar, but the resulting grain yield and protein yield differing significantly, the pulse system improved the fertilizer-N use efficiency for grain by 36.6% and enhanced the fertilizer-N use efficiency for protein by 62.6%, compared to the summerfallow systems. The cereal monoculture had a similar grain yield as the pulse system, but the grain yield from the monoculture system was associated with twice the amount of fertilizer used as the pulse system. Consequently, the pulse system enhanced the fertilizer-N use efficiency for grain by 99.0% and the fertilizer-N use efficiency for protein yield by 186.6%, compared to the cereal monoculture. The trend of the systems’ effect was shown across the years with average to above-average precipitation where the systems’ effect was similar to the effect that was observed in the dry years.

## Discussion

In rainfed dry areas, water is key for crop productivity^1^,^35^. Stored soil water plays an important role in seed germination, seedling establishment and the early stages of plant growth^30^, whereas rainfall during the crop growth period influences all the phases of plant development and crop yield^36^. Across the five cycles of the 3-yr cropping sequences in the present study, the soil retained a fairly large amount of water in the 0–1.2 m soil profile by crop harvest. A portion of this water is above the ‘permanent wilting point’ of water content for the particular soil^34^, suggesting that a portion of remaining water should be available for crops to be grown the following spring. The frost-free period averages 114.3 days over the 126 years of recorded data at the experimental site^25^, with the air temperature reaching below zero in the first and second week of September, and the soil starts freezing soon thereafter. Thus, the rather short growing season, which is typical of the northern latitude region of the North American Great Plains, might not allow some crops to have adequate time to utilize all of the water that is available during the growth period.

Rainwater during the growing season plays a much more important role in determining crop yield than pre-planting residual soil water^35^,^36^. Precipitation during the May-August crop growth period in our study totaled 194, 129, 270, 176 and 410 mm in 2006, 2007, 2008, 2009 and 2010, respectively. In these years, summerfallow conserved 48.5, 52.3, 52.1, 29.7 and −23.4 mm of water in the 0–1.2 m cm soil profile. Thus, approximately 79% of the precipitation during the growing season was not conserved by the summerfallow practice. The capacity of soil water storage and the ability of water use by crop plants can be a complex matrix with many factors involved^8^,^36^,^37^ such as evaporation (which is typically greater than 1500 mm annually at the experimental area) and evapotranspiration. However, the main difference between the summerfallow systems and the diversified system with pulses in terms of water use occurs during the period of 1 May to 31 August in Year-2 of the cropping sequence, where 79% of the un-conserved rainwater by the summerfallow system can be utilized for grain production through the adoption of the alternative, non-summerfallowing pulse systems.

One of the attractive features of summerfallowing is the release of N that is mineralized from soil organic matter during the summerfallow period^11^,^38^, and this N is then readily available to crops that are grown the subsequent year^11^. In the present study, we found that the pulse systems had soil N values at crop harvest that were 87% greater compared to the fields after a cereal (spring wheat or barley), with the pulse systems showing a similar effect on soil N as the summerfallow system. Noteworthy is that the quantity of soil N released during the summerfallow period varied substantially each year, suggesting that there is a high risk if crop production relies on the source of soil N that is released through summerfallow practices.

With pulse systems, the N-rich crop residue and roots decompose over the postharvest period, which provides N benefits to the crops the following year^39^ or even in the third year^32^. We found that the process of crop residue decomposition added additional mineral N to the soil N pools. By contrast, the summerfallow system lost soil N during the postharvest period, whereas the cereal monoculture systems had little or no change in postharvest soil N. The soil N dynamics are complicated^40^, and the quantity of soil N can change with many factors^41^,^42^. In the present study, the largest change in soil N over the postharvest period occurred in the fall 2007 to spring 2008 period when the summerfallow fields lost 63.9 N kg ha^−1^, whereas the fields after pulses increased soil N by 25.3 kg ha^−1^, and the fields after wheat increased by 18.5 kg ha^−1^. The amount of soil N remaining at harvest plus the amount of N contributed via straw and root decomposition during the fall and winter are readily available for the crops to be grown the following spring. Averaged across the five cycles, total N in the 0–1.2 m soil depth available to the Year-3 durum wheat, in the present study, was significantly higher in the fields following either the pulse crops or the summerfallow than in the fields after the cereal crops. Although the quantity of soil N available to the durum wheat was similar between the pulse- and summerfallow-based systems, the source of the N differed considerably. The increased available N in the pulse systems reflects the process by which the plants fix atmospheric N_2_ through symbiosis with soil *Rhizobium* and the decomposition of the N-rich crop residues, a biological process that is environmentally friendly^22^,^43^. By contrast, the increased soil N with summerfallow systems is mainly through ‘mining’ the soil and accelerating the depletion of the soil organic matter^23^, which is a soil-degrading and environmentally detrimental approach^44^.

One of the major goals of farming is to increase the crop yield per unit of input^45^,^46^. The present study shows that diversifying cropping systems with pulse crops can increase the total grain production by 35.5%, improve protein yield by 50.9%, and enhance fertilizer-N use efficiency for grain by 33.0% over the conventional summerfallow system. Although the durum wheat grown following the summerfallow in the rotation increased the grain yield by an average of 11.2% and enhanced the protein yield by 17.9% compared to the durum wheat grown following the pulses, the summerfallowing approach was unable to offset the opportunity loss of a grain crop during the summerfallow phase of the crop sequence. The production improvement with pulse systems was consistent regardless of the dry or the normal-to-wet conditions encountered in the present study. We also find that spring wheat or barley based cereal monoculture can produce a similar quantity of grain yield and protein yield as the pulse system, but the former system will require a significant amount of N fertilizer to achieve the level of grain yield. With pulse systems, the pulses are directly seeded between the rows of stubble that remain standing from the harvest of the previous crops. Standing stubble helps to improve the micro-environmental conditions that are beneficial for crop establishment in water-limited areas^47^. We argue that this direct seeding configuration is a significant improvement over the summerfallowing system where multiple tillage operations are typically used and little standing crop residue remains. The improved seeding configuration in combination with the positive attributes of pulse crops (i.e., utilizing rainwater and providing the N benefits determined in this study) provide a strong incentive for diversifying cropping systems with short-seasoned, shallow-rooted pulses as an effective alternative to conventional summerfallow systems in the dry areas of the northern latitudes.

The inclusion of annual pulses in farming systems, either as a green manure^48^,^49^ or grain crop^50^, has been shown to improve soil physical, chemical, and biological properties^51^, reduce soil degradation^38^, and enhance environmental sustainability^6^. Positive rotational effects of pulses to subsequent cereal or oilseed crops have been well documented in Mediterranean-type climates^52^. However, the findings on the rotational benefits of pulses in the short-season, semiarid northern latitudes have been inconsistent^49^,^53^,^54^,^55^,^56^. In particular, it is unclear in the scientific literature whether the beneficial features that are associated with conventional summerfallowing can be retained with improved, pulse-based systems in water scarce environments. The present study, which was based on five cycles of a 3-yr cropping sequence of field experiments, clearly demonstrates that this alternative, pulse-based approach is highly effective and productive in the short-season, semiarid northern latitude area.

The global demand for grains such as wheat is forecast to increase by 100–110% by 2050^57^ to meet the ever-growing human population’s need for food, feed, fiber and fuel. Given the limited availability of uncultivated farmland on the planet and the growing concerns over converting carbon-rich forests and grasslands to cropland^58^, most of the future increases in grain production will likely come from the existing farmland^45^. Thus, these alternative, pulse-based cropping systems, when used to diversify conventional summerfallow-based systems, can provide an opportunity to increase total grain production without exploring new farmland. More research could be needed, however, to quantify the potential environmental benefits that are associated with pulse-based cropping systems.

## Methods

### Experimental design

Field experiments were conducted at the Agriculture and Agri-Food Canada Research Centre near Swift Current, Saskatchewan (50°25′N, 107°44′W). The soil was an Aridic Haploboroll (Orthic Brown Chernozem in the Canadian soil classification) with a silt loam texture containing 28% sand, 49% silt, and 23% clay, and with an organic C content of 20 g kg^−1^ and pH (CaCI_2_) of 6.5 in the top 0.15-m depth at the beginning of the experiment (in 2005).

Five cycles of the 3-yr crop sequences were conducted from 2005 to 2011 (Table 1). The 1^st^ cycle was initiated in 2005 and was completed in 2007; the 2^nd^ cycle started in 2006 and was completed in 2008. Starting with cycle-3, we increased the number of pulse crops in the Year-2 crop mix (i.e., 2 dry pea cultivars, 2 chickpea cultivars, and 4 lentil cultivars, along with barley and summerfallow control). More pulses were added to test whether the different pulse cultivars act similarly when used to diversify cropping systems. In Year-1 of the cropping sequence, hard red spring wheat (cultivar AC Lillian) was no-till, direct seeded using the best crop management practices for wheat production in the local area^59^. At harvest, wheat stubble was cut to 15 cm high, and it was left standing; the rest of the plant residue was chopped and spread evenly across the plots. In Year-2 of the crop sequence, all crops were planted between the rows of the standing wheat stubble generated from Year-1. The pulse crops, along with a cereal (spring wheat in the first 2 cycles and barley in the latter 3 cycles) and a summerfallow control, were arranged using a randomized complete block design with four replicates. In Year-3 of the cropping sequence, durum wheat (cultivar AC Strongfield) was uniformly planted in each of the Year-2 plots.

### Planting and plot management

All plots were planted using a no-till plot seeder that was equipped with fertilizer, inoculant, and seed boxes, thus allowing for the application of fertilizers and seed (and *Rhizobium* inoculants for pulses) in a one-pass operation without pre-planting tillage. Plots were planted 60 mm deep with a row spacing of 0.228 m. The plot size was 2 m × 8 m. The planting was completed in the first to second week of May, and it varied slightly each year (Table 1). The seed rates for all crops were determined based on pre-seeding germination tests, seed size, and an estimated field emergence rate, targeting an optimal plant density for each crop.

For the Year-2 pulse crops, pre-packaged seeds and granular *Rhizobium* inoculants were evenly distributed in the eight-seed rows in each plot. The Year-2 cereal crops were fertilized with urea (CH_4_N_2_O containing 46% of N) at the soil test recommended rates (Table 1). All cropped plots (cereals and pulses) were fertilized with monoammonium phosphate (NH_4_H_2_PO_4_ containing 11% of N and 51% of P_2_O_5_), side-banded to a 30 mm distance from the seed-rows and 70 mm deep, to provide a total P (soil P + fertilizer P) of 27.5 P kg ha^−1^. No additional N-fertilizer was applied to the pulse crops.

For Year-3 durum wheat, the seed was treated with *Vitaflo* (e.g., 15.6% carbathiin and 13.2% thiram formulated as a liquid suspension) at a rate of 2.6 ml kg^−1^ of seed. All plots were fertilized with monoammonium phosphate to reach a level of 27.5 P kg ha^−1^. To quantify the N credits from the previous pulse crops and summerfallow, no additional N-fertilizer was applied to durum wheat except the N portion from the monoammonium phosphate. Weeds were controlled with 200 g a.e. ha^−1^ of glyphosate [N-(phosphonomethyl)glycine] prior to planting with no in-crop pesticide applied.

For the summerfallow control plots in Year-2, commonly-used practices were employed for weed management during the summerfallow period^59^ consisting of three to four tillage operations using a cultivator and rodweeder. The crops grown on summerfallow were managed using the same practices as were used for the crops grown on stubble.

### Data collection

Each year, soil samples were taken within 3 days prior to planting and again within 3 days after crop harvest. Two 30-mm diameter soil cores (with core samples kept separate) were taken to the depth of 1.2 m in each plot. Crops grown in this semiarid northern latitude area typically do not root below a depth of 1.2 m^29^. Each of the two cores was divided into 0–0.15, 0.15–0.30, 0.30–0.60, 0.60–0.90, and 0.90–1.20 m increments. These soil samples were analyzed for soil nutrients and water using standard procedures adopted at the Agriculture and Agri-Food Canada Research Centre Soil Chemistry Service Laboratory^35^,^48^,^59^. The available soil N (NO_3_^−^ + NH_4_^+^) was determined using the Kjeldahl nitrogen digestion method with sulfuric acid and a metal catalyst, and the soil water content was measured using an oven-dry method. The measured values were converted to volumetric units using soil bulk densities of 1.16, 1.29, 1.39, 1.54 and 1.63 g cm^−3^, respectively, for the five soil depths^35^,^59^. The analytic results of the two cores in each plot were averaged to provide the plot value for statistical analysis.

The total amount of water used by a crop was calculated as the difference in soil water content between planting and harvest sampling dates plus the precipitation received during the growing season with negligible deep drainage^36^. The soil water measurements taken at harvest determined the amount of soil water above the permanent wilting point but unused by the current crops, whereas the soil water content measured at spring planting time provides an indication of how much recharge water has been added to the soil profile during the fall and winter months. At full maturity (the specific dates are presented in Table 1), the center six plant rows in each plot were harvested with a plot combined for the determination of grain yield. The plants in a 1.0 m^2^ area in each plot were hand-harvested for the determination of the biomass of grain and straw. The N concentrations in the grain and straw were analyzed using NIR method^59^.

During the five cycles of the field experiment, the precipitation in 2007 and 2009 was lower than the long-term average, whereas the precipitation in 2006, 2010 and 2011 was near or above the long-term average (Fig. 2); they were categorized as ‘dry’ and ‘normal-to-wet’, respectively. To evaluate the productivity of the diversified systems with pulses in different growing conditions, we calculated the total grain yield of the 3-yr cropping sequences for the dry and normal-to-wet categories (Table 5). For example, in the drier years of 2007 and 2009, Year-1 (spring wheat) occurred in cycles-3 and -5, respectively; Year-2 (pulses, summerfallow, cereal) occurred in cycles-2 and -4, respectively; and Year-3 (durum wheat) occurred in cycles-1 and -3, respectively (Table 1). The yield of the wheat-cereal-durum wheat rotation under dry conditions was calculated as the average of the Year-1 spring wheat yields for 2007 and 2009, the average of the Year-2 cereal yields and the average of the Year-3 durum wheat yields. The same approach was used to calculate the yields of the wheat-pulse-durum wheat and the wheat-summerfallow-durum wheat systems. Using this method, the total quantity of grain produced over each 3-yr cropping sequence was calculated and compared, giving an overview of the effectiveness and productivity of diversifying cropping systems with pulse crops in comparison with the other systems. Although this approach does not recognize differences in the value of the grains, it does provide an assessment of the overall productivity for each of the systems.

Weather data were collected from the weather station located on the Research Farm, approximately 500 meters from the plot site. The precipitation and air temperature (max. and min.) data are presented in Fig. 2.

### Statistical analysis

All data were analyzed with mixed models using the MIXED procedure of SAS^60^, with previous crop and summerfallow as fixed effects, and block and interactions as random effects. For variables showing a significant interaction between the applied treatments and the cycle of the experiment, the treatment effects were discussed separately for each cycle. For variables where treatment effects followed similar trends among the cycles, the values were pooled together across the cycles, and the overall means are presented.

## Additional Information

**How to cite this article**: Gan, Y. *et al.* Diversifying crop rotations with pulses enhances system productivity. *Sci. Rep.* **5**, 14625; doi: 10.1038/srep14625 (2015).

## Figures and Tables

**Table 1:** Basic agronomic information for crops in each of the five cropping cycles.

Cycle	Croppingsequence	Calendaryear	Crop	Cultivar	N-P-K-S (kg ha^−1^)		Seeding rate(#seeds m^−2^)	Date/month of		Growthperiod (d)
					Pre-seeding soilnutrient	Fertilizerapplied		Seeding	Mature	
1	Yr-1	2005	Spr. wheat	AC-Lillian	24-21-380-25	62.5-22.7-0-0	250	02-May	25-Aug	115
	Yr-2	2006	Dry pea	Golden	24-23-450-24	5.9-27.5-0-0	90	05-May	02-Aug	89
			Lentil	Glamis	24-23-450-25	5.9-27.5-0-0	140	05-May	14-Aug	101
			Spr. wheat	AC-Lillian	24-23-450-26	68.2-22.7-0-0	250	05-May	26-Aug	113
	Yr-3	2007	Dur. wheat	AC-Strongfield	Varied^a^	5.9-27.5-0-1	250	08-May	28-Aug	112
2	Yr-1	2006	Spr. wheat	AC-Lillian	24-23-450-24	68.2-22.7-0-0	250	05-May	06-Aug	93
	Yr-2	2007	Dry pea	Golden	20-25-452-49	5.9-27.5-0-0	90	08-May	18-Jul	71
			Lentil	Glamis	20-25-452-50	5.9-27.5-0-0	140	08-May	21-Jul	74
			Spr. wheat	AC-Lillian	20-25-452-51	45.0-27.5-0-0	250	08-May	18-Aug	102
	Yr-3	2008	Dur. wheat	AC-Strongfield	Varied^a^	5.9-27.5-0-1	250	13-May	02-Sep	112
3	Yr-1	2007	Spr. wheat	AC-Lillian	20-25-452-51	45.0-27.5-0-0	250	08-May	18-Aug	102
	Yr-2	2008	2 Dry pea	Golden, Handel	26-23-388-32	5.9-27.5-0-0	90	07-May	10-Aug	95
			2 Chickpea	Vanguard, Frontier	26-23-388-33	5.9-27.5-0-0	60	07-May	24-Sep	140
			4 Lentil	Glamis, Richlea, Robin, Impact	26-23-388-34	5.9-27.5-0-0	140	07-May	15-Aug	100
			Barley	Metcalfe	26-23-388-34	45.0-27.5-0-0	250	07-May	16-Aug	101
	Yr-3	2009	Dur. wheat	AC-Strongfield	Varied^a^	5.9-27.5-0-1	250	04-May	28-Sep	147
4	Yr-1	2008	Spr. wheat	AC-Lillian	26-23-388-34	45.0-27.5-0-0	250	07-May	21-Aug	106
	Yr-2	2009	2 Dry pea	Golden, Handel	9-28-481-18	5.9-27.5-0-2	90	12-May	10-Aug	90
			2 Chickpea	Vanguard, Frontier	9-28-481-19	5.9-27.5-0-1	60	12-May	22-Sep	133
			4 Lentil	Glamis, Richlea, Robin, Impact	9-28-481-20	5.9-27.5-0-0	140	12-May	12-Aug	92
			Barley	Metcalfe	9-28-481-21	63.8-27.5-0-1	250	12-May	21-Aug	101
	Yr-3	2010	Dur. wheat	AC-Strongfield	Varied^a^	5.9-27.5-0-1	250	14-May	28-Sep	137
5	Yr-1	2009	Spr. wheat	AC-Lillian	9-28-481-21	63.8-27.5-0-1	250	12-May	31-Aug	111
	Yr-2	2010	2 Dry pea	Golden, Handel	22-34-326-51	5.9-27.5-0-2	90	14-May	04-Aug	82
			2 Chickpea	Vanguard, Frontier	22-34-326-52	5.9-27.5-0-1	60	14-May	06-Sep	115
			4 Lentil	Glamis, Richlea, Robin, Impact	22-34-326-53	5.9-27.5-0-0	140	14-May	14-Aug	92
			Barley	Metcalfe	22-34-326-54	63.8-27.5-0-0	250	14-May	25-Aug	103
	Yr-3	2011	Dur. wheat	AC-Strongfield	Varied^a^	5.9-27.5-0-1	250	29-Apr	14-Sep	138

The 3-yr cropping sequences were run for five cycles at Swift Current, Saskatchewan, Canada, 2005–2011.

^a^Depending on crops grown the previous year.

**Table 2:**
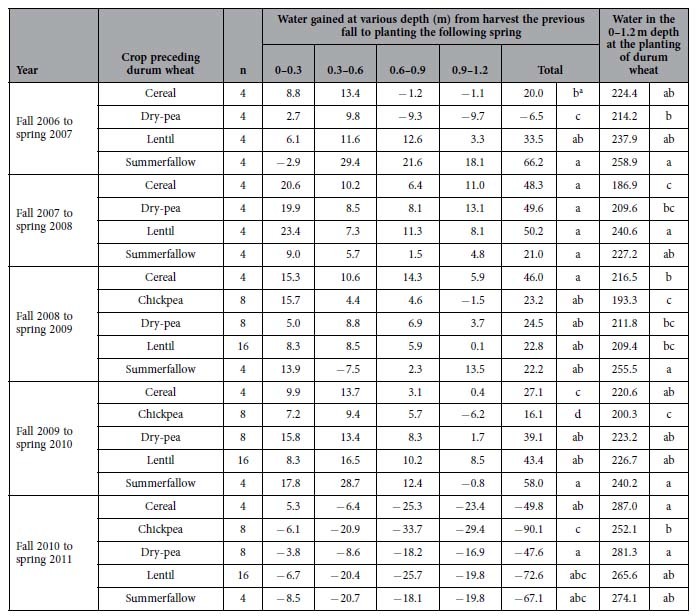
Soil water (mm) in different cropping systems.

Soil water recharged at various depths was calculated as the soil water at planting time the following spring subtracted by the water remaining at harvest the previous fall.

^a^Significance between preceding crops (or summerfallow) within a year at *P* < 0.05.

**Table 3:**
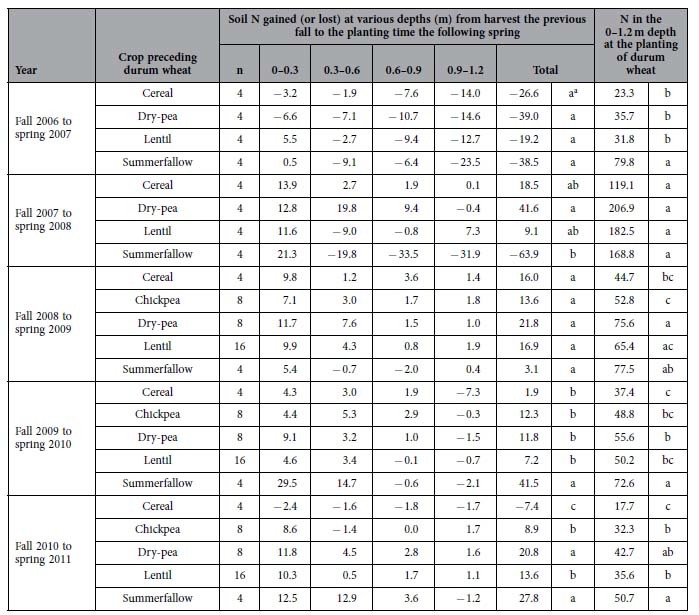
Soil N (N kg ha^−1^) in different cropping systems.

Soil N gained (or lost) at various depths was calculated as soil N in the spring planting time subtracted by the soil N remaining at harvest the previous fall.

^a^Significance between preceding crops (or summerfallow) within a year at *P* < 0.05.

**Table 4:**
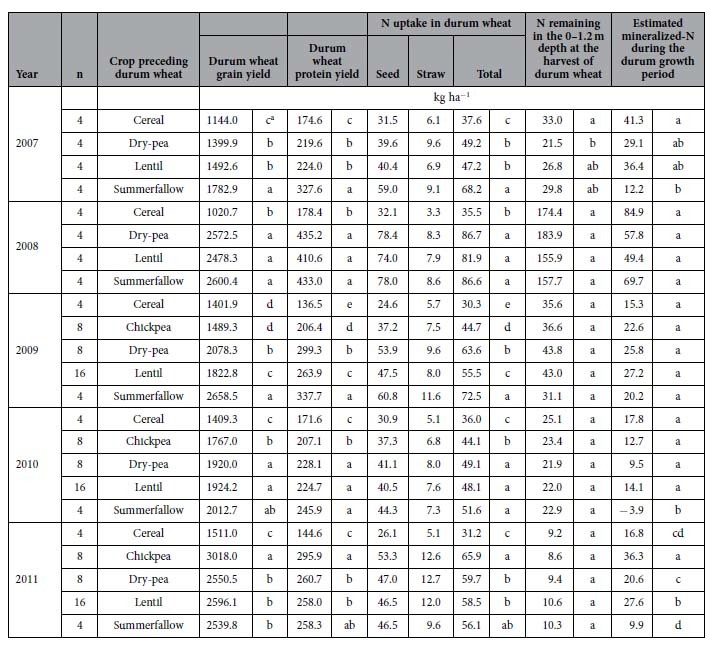
Durum wheat yield and N dynamics in different cropping systems.

Durum wheat was preceded by different crops or summerfallow in the 3-yr cropping sequence conducted for five cycles at Swift Current, Saskatchewan.

^a^Significance between preceding crops (or summerfallow) within a year at *P* < 0.05.

**Table 5:** Productivity of different cropping systems under dry and normal-to-wet categories.

System	Grainyield	Proteinyield	FertilizerN applied	Fertilizer-N useefficiency	
				Grain	Protein
Dry	kg ha^−1^			kg ka^−1^ of N	
** a**, wheat-fallow-durum	4312.9	624.2	59.7	74.9	10.8
** b**, wheat-pulse-durum	5895.9	1004.9	58.2	102.4	17.5
** c**, wheat-cereal-durum	5561.2	664.4	116.3	51.5	6.1
Comparison between systems					
** b** over **a** (%)	36.7	61.0	−2.4	36.6	62.6
** c** over **a** (%)	28.9	6.4	94.9	−31.3	−43.3
** b** over **c (%)**	6.0	51.2	−49.9	99.0	186.6
Normal to wet					
** a**, wheat-fallow-durum	4720.4	654.3	64.9	74.8	10.3
** b**, wheat-pulse-durum	6341.2	921.0	66.8	96.7	14.0
** c**, wheat-cereal-durum	6302.7	709.4	122.4	51.8	5.9
Comparison between systems					
** b** over **a** (%)	34.3	40.7	2.9	29.3	35.7
** c** over **a** (%)	33.5	8.4	88.6	−30.7	−43.1
** b** over **c (%)**	0.6	29.8	−45.5	86.6	138.7

The values are the sum of the crops grown in the 3-yr cropping sequence, averaged over the five cycles.
